# Outcomes of HIV pregnant women on Option B+ treatment in an urban Malawian private medical clinic, between July 2011 and November 2013

**DOI:** 10.1186/1471-2334-14-S2-P82

**Published:** 2014-05-23

**Authors:** Sekeleghe Kayuni

**Affiliations:** 1 MASM Medi Clinics Limited, Blantyre, Malawi

## Background

Malawi is one of the sub-Saharan African countries adversely affected by HIV/AIDS. Option B+ was adopted by Malawi from 1st July 2011 as an effective way of managing HIV through lifelong ART in pregnancy. Poor ART outcomes such as high default rates have been reported, raising concerns of setbacks on PMTCT progress. We assessed the outcomes of HIV infected pregnant clients on Option B+ at ART clinic at an urban Private medical facility in Malawi.

## Materials and methods

This retrospective survey was conducted in December 2013 at Kanjedza Medi Clinic. All records of Option B+ clients between July 2011 and November 2013 were reviewed. Information on age, HIV test, ART initiation and duration, adverse reactions, ART outcome (alive on ART, defaulted, dead, stopped, transfer out), delivery status and baby HIV test were analyzed.

## Results

There were 1,253 clients registered in the ART clinic and 711 alive, taking ART as of 30th November 2013. Fifty-one clients (pregnant women) tested HIV positive and registered on Option B+ after adherence counseling. 9 clients transferred out, remaining with 42 clients in the clinic register.

Forty clients (78.4% of all Option B+ clients and 95.2% of remaining clients) were alive and on ART. Their age range was 23 to 55 years old, with mean of 31.6 years (confidence interval CI; 24.4-39.4). ART duration ranged from 33 days to 794 days, with mean of 428 days. No adverse reactions were reported. Two clients (3.9%) defaulted ART after 182 days and 183 days respectively.

27 clients delivered during the period with 1 baby dying within an hour of birth. 18 babies were tested at 6 months of age and all were HIV negative. One client who defaulted delivered a baby who tested negative at age of 6 months.

## Conclusion

We noted better ART outcomes including lower default rate in our Option B+ clients. Babies were tested HIV non-reactive, depicting the success of Option B+ treatment. High default rates can be addressed through Option B+ program strengthening, adherence counseling and continued follow-up of clients.

